# Gut Microbiota-Mediated Drug-Drug Interaction between Amoxicillin and Aspirin

**DOI:** 10.1038/s41598-019-52632-5

**Published:** 2019-11-07

**Authors:** Juanhong Zhang, Yuemei Sun, Rong Wang, Junmin Zhang

**Affiliations:** 1 Key Laboratory for Prevention and Remediation of Plateau Environmental Damage, 940th Hospital of Joint Logistics Support Force of CPLA, Lanzhou, 730050 China; 2 School of Pharmacy, Lanzhou University, Lanzhou, 730000 China

**Keywords:** Drug delivery, Drug development

## Abstract

The effects of antibiotics on the intestinal flora can create potential drug-drug interactions. The combination of amoxicillin and aspirin is high and there is a high probability of interaction. We used 16S rRNA, incubation experiments and liquid chromatography-tandem mass spectrometry to analyze rat biological samples to characterize the effect of amoxicillin on the pharmacokinetics of aspirin metabolites. We first discovered that amoxicillin reduced the species and number of intestinal flora in rats, such as reducing the abundance of *Helicobacter pylori* and *Prevotella_copri*. After 12, 24, and 36 hours of incubation, the remaining amount of aspirin in the aspirin and amoxicillin treatment groups decreased, and salicylic acid production increased, suggesting that aspirin is metabolized by the intestinal flora, and the main metabolite is salicylic acid. As the incubation time prolonged, the reduction of aspirin and the production of salicylic acid in the amoxicillin treatment group were slower. It is indicated that the metabolic activity of aspirin through the intestinal flora is slowed down after administration of amoxicillin. The pharmacokinetic experiments showed that after administration of amoxicillin, the area under the salicylic acid curve increased by 91.38%, the peak concentration increased by 60.43%, and the clearance rate decreased by 43.55%.The results demonstrated that amoxicillin affected the pharmacokinetics of aspirin active metabolite salicylic acid by slowing down the metabolic activity of intestinal flora on aspirin. The interaction between amoxicillin and aspirin mediated by the intestinal flora may affect the efficacy of aspirin and cause more significant adverse effects.

## Introduction

The gut microflora is a large and diverse micro-ecological system, and the gut microbes maintain a dynamic balance between the organism and the body^1^. However, the external environment, pathological state of the body, and xenobiotics may cause dysbacteriosis, trigger various diseases, and even affect the metabolism and absorption of drugs, thereby affecting the clinical therapeutic effect^2–4^. Antibiotics can alter the intestinal flora, allowing bioconversion of other drugs used together before absorption in the gastrointestinal tract^5^, which means that the effects of antibiotics on the intestinal flora produce potential drug-drug interactions (DDI).

Aspirin is a salicylic acid derivative, an antipyretic, analgesic, anti-inflammatory and antithrombotic drug that is rapidly absorbed after oral administration^6^. Amoxicillin is also widely used as a clinically used antibiotic^7^, and has a higher probability of being combined with aspirin. Aspirin is reported to be metabolized by the intestinal flora when passing through the gastrointestinal tract, and ampicillin affects the antithrombotic effect of aspirin by altering the intestinal flora^8^. Based on the above background, we reasoned that the question: Does amoxicillin affect the biotransformation of aspirin by altering the metabolic activity of the intestinal flora? Does it affect the *in vivo* pharmacokinetics of aspirin? In order to solve the above problems, this experiment uses chromatographic techniques and microbiological analysis methods to study the interaction between amoxicillin and aspirin in pharmacokinetics, and provide experimental basis for clinical rational drug use.

## Results

### Methodological investigation

**Figure 1:**
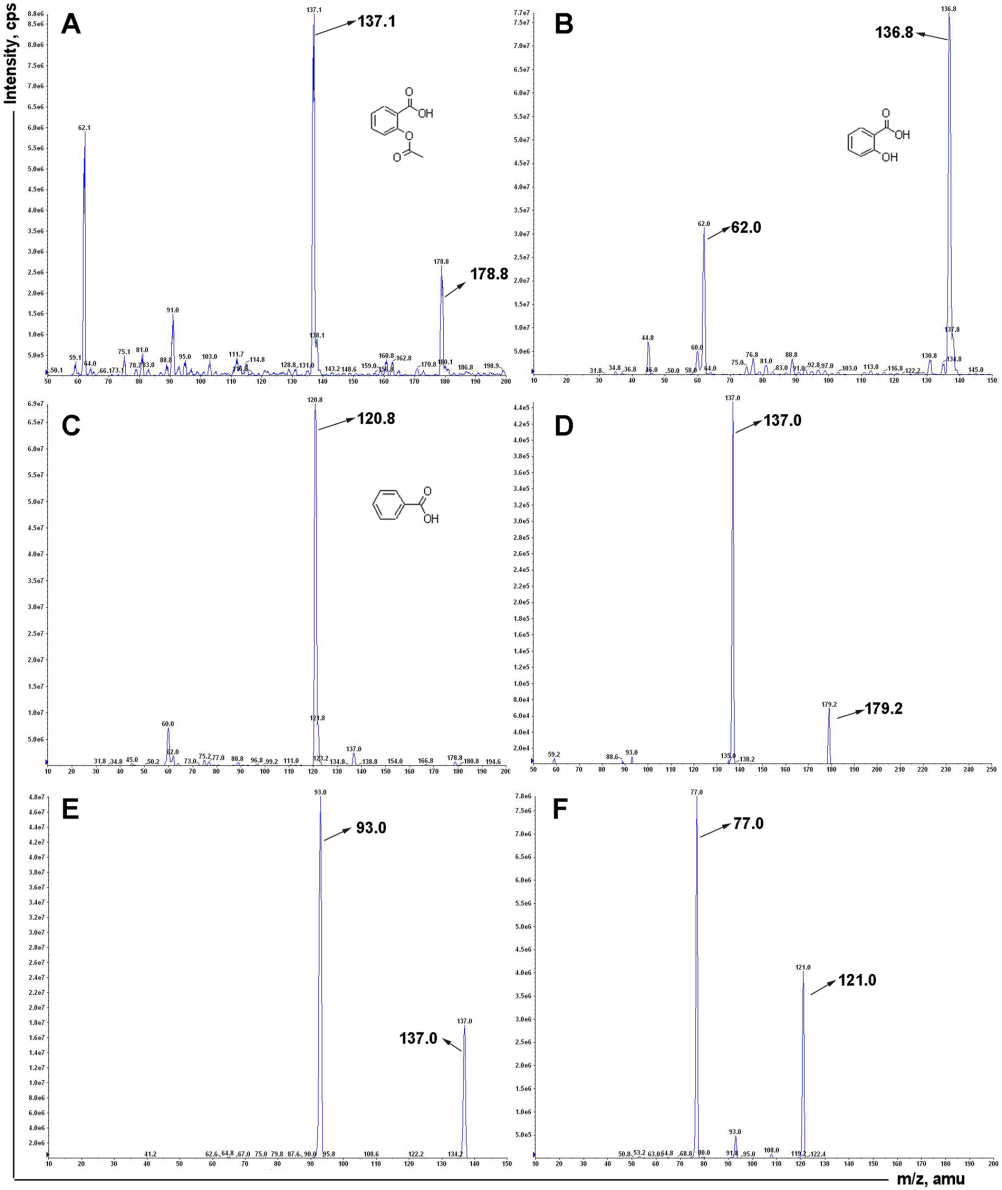
The mass spectrum of (**A**) parent ion scan of aspirin, (**B**) parent ion scan of salicylic acid, (**C**) parent ion scan of benzoic acid, (**D**) fragment ion scan of aspirin, (**E**) fragment ion scan of salicylic acid, (**F**) fragment ion scan of benzoic acid.

**Figure 2:**
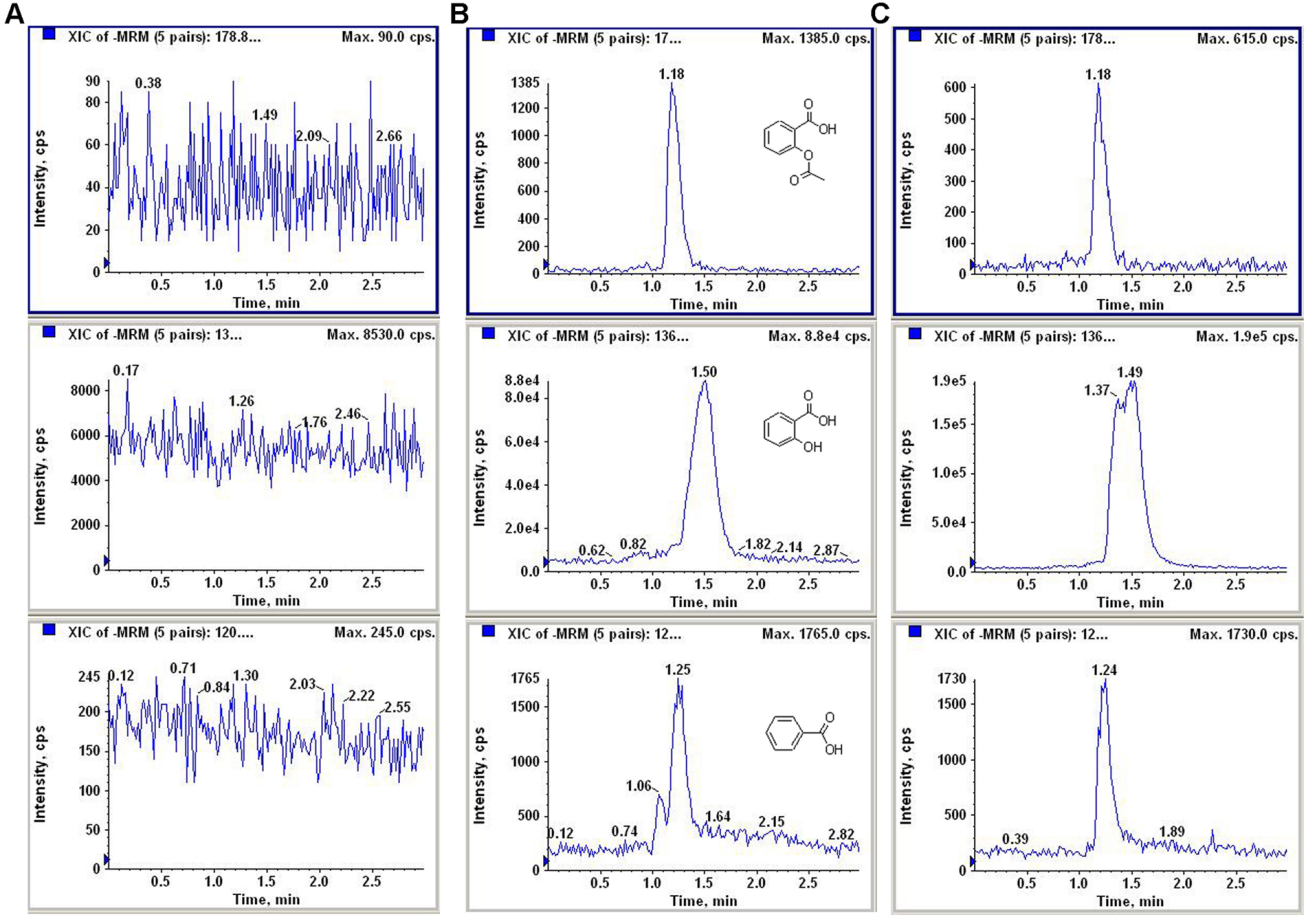
Chromatograms of aspirin and salicylic acid of (**A**) blank plasma, (**B**) blank plasma spiked with standard aspirin and salicylic acid and (**C**) sample.

A method for simultaneous determination of aspirin and salicylic acid in biological samples by LC-MS/MS was established. The primary and secondary scans of aspirin, salicylic acid and internal standard benzoic acid mass spectra are shown in Fig. 1A–F. The detected ion pairs of aspirin, salicylic acid and internal standard benzoic acid were *m/z* 178.8 → 137.0, *m/z* 136.8 → 93.0, *m/z* 120.8 → 77.0, respectively. The collision induced dissociation voltages were −11 psi, −26 psi, and −24 psi, respectively. The collision energy is −13 eV, −26 eV, and −18 eV. The mass spectrometry spray voltage was −3500 V and the atomization temperature was 250 °C. The mobile phase was acetonitrile-water-formic acid (90:10:0.1, v/v/v) and each sample was analyzed for 3.0 minutes. Assays for aspirin and salicylic acid were analyzed for blank fecal fluid samples, aspirin and salicylic acid-containing fecal fluid samples from rat fecal samples (Fig. 2A–C). The retention times of aspirin and salicylic acid were 1.18 and 1.50 min, respectively. Impurities do not interfere with sample determination.

### The effect of amoxicillin on intestinal flora

**Figure 3:**
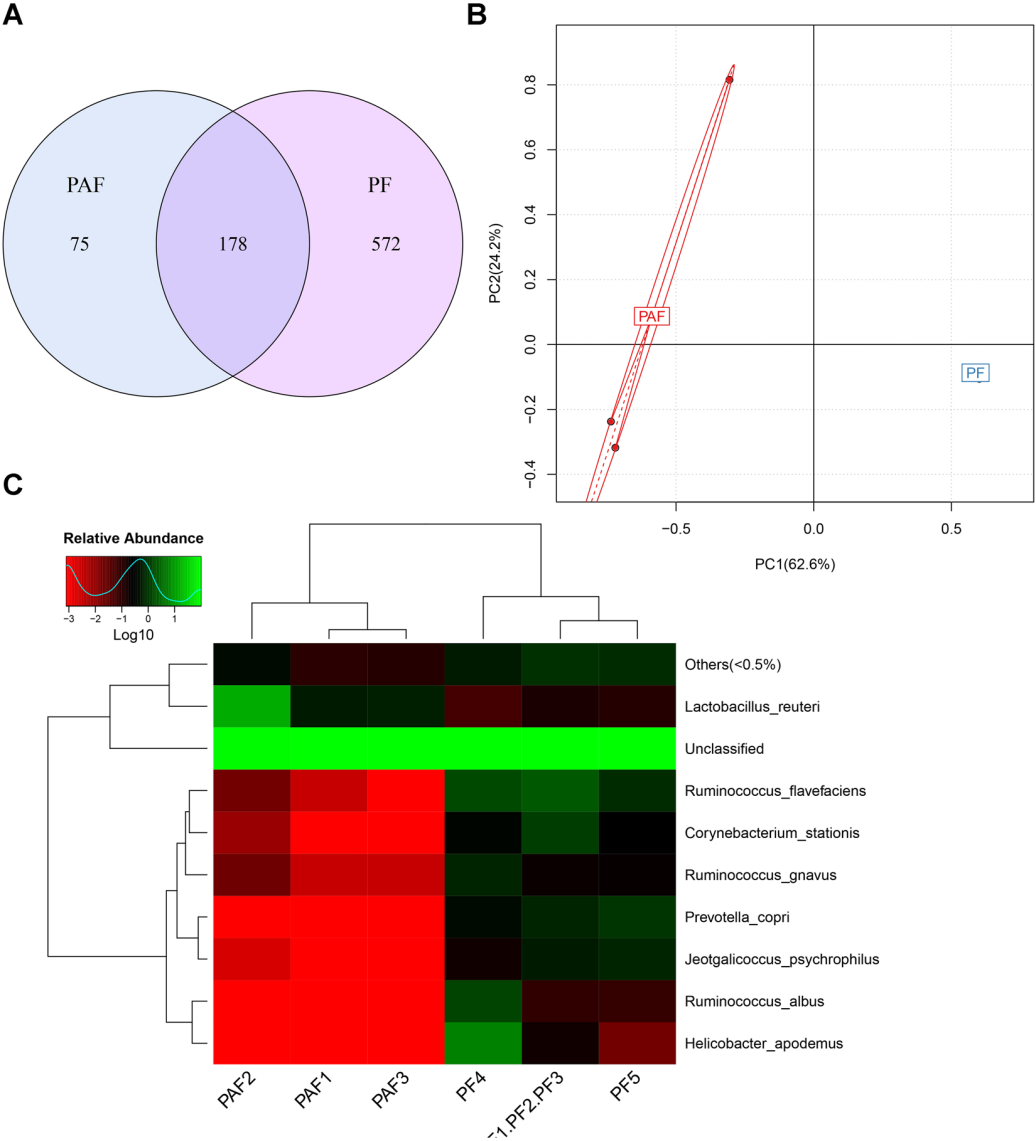
16 S rRNA analysis results of (**A**) venn diagram, (**B**) principal component analysis and (**C**) log-scaled percentage heat map of species level. PF: Fecal samples of aspirin group (N1), PAF: Fecal samples of amoxicillin treatment group (N2).

Currently, a more advanced and reliable method for monitoring changes in bacterial flora is 16S rRNA^4^. To characterize the effects of amoxicillin on the gut microbiota, we analyzed the changes in the aspirin group (N1) and the combination of amoxicillin and aspirin group (N2) using the 16S rRNA method. As can be seen from the venn analysis in Fig. 3A, the number of species in the aspirin group was as high as 750, while the number of species in the amoxicillin-treated group was only 253, and the number of species in the amoxicillin group was significantly reduced. In the principal component analysis of Fig. 3B, the distribution distances of the two sets of samples are far apart and there are differences. The heat map analysis of Fig. 3C shows a difference in the abundance of certain species between the two groups. The data in Fig. 3 demonstrates that amoxicillin reduces the abundance of the intestinal flora in rats. The more obvious reductions are *Helicobacter_apodemus* and *Prevotella_copri*.

### The effect of amoxicillin on the metabolism of aspirin

**Figure 4:**
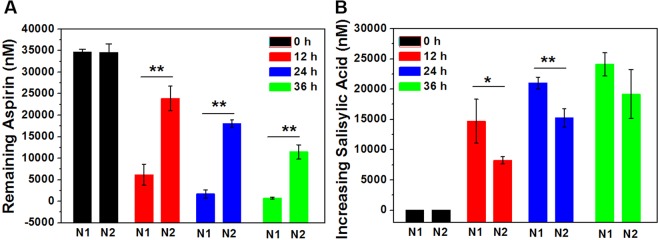
Drug concentration of the incubation system of (**A**) amount of remaining aspirin and (**B**) the amount of salicylic acid produced in N1 and N2 group. Note: ^*^P < 0.05, ^**^P < 0.01, compared with N1 group.

After demonstrating that amoxicillin affects the intestinal flora, we immediately observed the effect of amoxicillin on the metabolism of aspirin through the intestinal flora. After aspirin was incubated with rat fecal extract for 0, 12, 24, and 36 hours, the remaining amount of aspirin and the resulting salicylic acid were determined by LC-MS/MS. As shown in Fig. 4A,B and Table 1, after incubation for 12 h, aspirin in N1 group and N2 group decreased by 28438.33 and 10666.67 nM, respectively, and the salicylic acid production amounts were 14700.00 and 8248.33 nM, respectively. The amount of aspirin and salicylic acid in the two groups varied significantly.

**Table 1:** Changes in the content of aspirin and salicylic acid in N1 and N2 groups in the incubation system.

Time (h)	Amount of remaining aspirin (nM)		Amount of salicylic acid produced (nM)	
	N1	N2	N1	N2
0	34600.00 ± 736.55	34550.00 ± 1983.05	0.00	0.00
12	6161.67 ± 2444.07	23883.33 ± 2859.34^**^	14700.00 ± 3631.46	8248.33 ± 581.47^*^
24	1686.83 ± 990.40	17983.33 ± 894.89^**^	21000.00 ± 996.24	15250.00 ± 1540.29^**^
36	731.67 ± 224.45	11460.00 ± 1647.21^**^	24100.00 ± 1925.49	19183.33 ± 4046.71

^*^P < 0.05, ^**^P < 0.01, compared with N1 group.

Overall, after 12, 24, and 36 hours of incubation, the remaining amount of aspirin in the N1 and N2 groups decreased, and salicylic acid production increased, indicating that aspirin was metabolized by the intestinal flora, and the main metabolite was salicylic acid. As can be seen from Fig. 4A and Table 1, as the incubation time prolonged, the reduction in aspirin in the N2 group was slower than in the N1 group. In Fig. 4B and Table 1, the amount of salicylic acid produced in the N1 and N2 groups increased, and the N2 group increased more slowly than the N1 group. The results showed that the metabolism of aspirin in the intestinal flora of rats was slowed down after treatment with amoxicillin.

### Pharmacokinetics of aspirin

**Figure 5:**
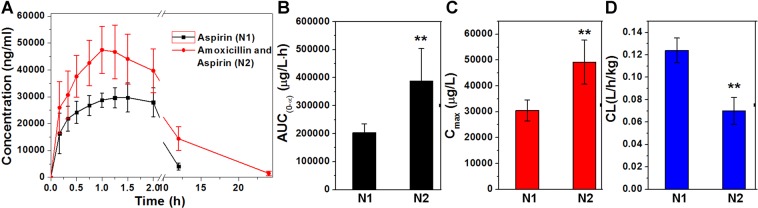
The pharmacokinetic characteristics of salicylic acid of (**A**) concentration-time curve, (**B**) the area under the curve, (**C**) the peak concentration and (**D**) clearance rate in N1 and N2 groups. ^**^P < 0.01, compared with N1 group.

After demonstrating that amoxicillin could attenuate the metabolism of aspirin through the intestinal flora, we are very interested in the plasma concentration of aspirin and the active metabolite salicylic acid. After a single dose of aspirin tablets in rats, the plasma concentration was determined by LC-MS/MS. Since aspirin was metabolized faster, salicylic acid was used to determine the amount of aspirin absorbed into the blood. The drug concentration-time curves were shown in Fig. 5A. The area under the curve of N1 and N2 groups (AUC_(0-t)_) were (203923.45 ± 30938.31) and (390263.26 ± 113845.08) µg·L^−1^·h^−1^ (Fig. 5B and Table 2), the peak concentration (C_max_) was (30533.33 ± 4013.83) and (48985.39 ± 8702.18) µg·L^−1^ (Fig. 5C and Table 2), clearance rate (CL) were (0.124 ± 0.01) and (0.07 ± 0.01) L·h^−1^·kg^−1^ (Fig. 5D and Table 2), respectively. It was found that after administration of amoxicillin, AUC_(0-t)_ increased by 91.38%, C_max_ increased by 60.43%, and CL decreased by 43.55%. It is indicated that amoxicillin affects the pharmacokinetics of aspirin active metabolite salicylic acid by slowing down the metabolism of intestinal flora.

**Table 2:** Changes in pharmacokinetic parameters (AUC_(0-t)_, C_max_ and CL) of salicylic acid in N1 and N2 groups.

Parameters	N1	N2
AUC_(0-t)_ (ug/L*h)	203923.45 ± 30938.31	390263.26 ± 113845.08^**^
C_max_ (ug/L)	30533.33 ± 4013.83	48985.39 ± 8702.18^**^
CL (L/h/kg)	0.124 ± 0.01	0.07 ± 0.01^**^

^**^P < 0.01, compared with N1 group.

## Discussion

The intestinal flora plays a significant role in the metabolism of exogenous substances and drugs^9,10^. It is worth noting that oral medications come into contact with the intestinal flora before entering the bloodstream and are likely to be transformed by the gut flora, resulting in decreased therapy activity or increased toxic metabolites. These results directly affect the amount of the original drug and metabolites entering the blood circulation, and ultimately changes the bioavailability and efficacy of the drug. Of course, the intestinal flora is also a relatively fragile “invisible organ”. For example, the use of antibiotics is very likely to cause changes in the flora, which in turn affects the efficacy of the combination of drugs^11,12^. In this experiment, the results of 16S rRNA demonstrated that after administration of amoxicillin, the intestinal flora of rats could change significantly (Fig. 3), such as reducing the abundance of *Helicobacter_apodemus* and *Prevotella_copri*, and the significant changes may be directly related to drug metabolism and pharmacokinetics.

We have confirmed the effects of the intestinal flora on aspirin metabolism in line with previous report^8^. It is further shown that changes in the composition of the intestinal flora by amoxicillin could cause changes in the metabolic activity of aspirin. The results of the incubation experiment showed that the activity of aspirin metabolism in the intestinal flora was weakened after administration of amoxicillin (Table 1 and Fig. 4), which means that the biotransformation of aspirin before absorption was reduced, so that the amount of aspirin staying in the intestinal tract would increase. The situation is highly likely to result in an increase in the amount of aspirin absorbed into the blood.

Since aspirin is rapidly metabolized and almost undetectable in plasma, we further confirmed that amoxicillin can affect the pharmacokinetics of aspirin through the pharmacokinetic changes of its active metabolite salicylic acid in rats. The results showed that after administration of amoxicillin, the absorption of aspirin in the blood increased, the amount of metabolite salicylic acid increased, and the AUC_(0-t)_ increased significantly (Table 2 and Fig. 5), indicating that amoxicillin affects the pharmacokinetic properties of aspirin. Pharmacokinetic characteristics are an important theoretical basis for rational drug use, so changes in pharmacokinetic parameters will undoubtedly affect the therapeutic effect of drugs.

Amoxicillin and aspirin are commonly used as clinical drugs, and there are many possibilities for DDI in the treatment process. Currently, there are few studies on DDI caused by intestinal flora, and most studies on DDI focus on metabolic enzymes and transporters^13–15^. In order to prevent DDI caused by changes in metabolic enzymes and transporters, this study administered amoxicillin three days before the pharmacokinetic experiments, allowing amoxicillin to act on the intestinal flora. After excretion of amoxicillin, aspirin was administered to study DDI caused by intestinal flora. In general, when antibiotics are combined with drugs that are metabolized by the intestinal flora, the interaction of the pharmacokinetics mediated by the intestinal flora should be taken seriously, which may be one of the most important factors affecting the efficacy of the drug. It is worth noting that our results were limited to the rat model, but failed to detect changes in human flora and pharmacokinetic parameters of aspirin and amoxicillin co-treatment. These further clinical evidence require us to proceed.

## Conclusions

In conclusion, amoxicillin affects the pharmacokinetic profile of the aspirin-active metabolite salicylic acid by slowing the metabolic activity of aspirin through the intestinal flora. The interaction between amoxicillin and aspirin mediated by the intestinal flora may affect the efficacy of aspirin and cause more significant adverse effects.

## Materials and Methods

### Instruments and reagents

High performance liquid chromatography (UFLC-20A, Shimadzu Corporation, Japan); Shim-pack XR-ODS column; Triple quadrupole mass spectrometer (API3200, Applied Biosystems, USA). Amoxicillin capsules (batch number 1706011) were provided from Shandong Lukang Pharmaceutical Co., Ltd. Aspirin tablets (batch number BJ34684), aspirin reference substance (batch number: 100113-201104), salicylic acid reference substance (batch number: J22S6J3607), and benzoic acid reference substance (batch number 2016-2003) were purchased from China National Institute for the Control of Pharmaceutical and Biological Products.

### Animal experiment

Twelve wistar rats weighing approximately 200 g (license number: SCXK (Gan) 2015-0001) were provided by the Lanzhou Veterinary Research Institute of the Chinese Academy of Agricultural Sciences. Rats were randomly divided into the aspirin group (N1) and the amoxicillin and the aspirin combination group (N2). The N1 group collected blank feces and quickly stored the feces in liquid nitrogen. After 12 hours of fasting, blank blood was collected, then aspirin (37.5 mg·kg^−1^) was administered and the blood sample was collected from the orbital venous plexus to a volume of approximately 0.3 mL. The blood sample collection time was 0.17, 0.33, 0.5, 0.75, 1, 1.5, 2, 4 6, 8, 12, 24 hours. Whole blood was centrifuged at 4000 r·min^−1^ for 5 minutes, the upper plasma was aspirated and stored at −80 °C. In the N2 group, amoxicillin (157.5 mg·kg^−1^) was orally administered once a day for three consecutive days prior to the pharmacokinetic experiment. On the second day after the end of the administration of amoxicillin, fasting was performed. On day three, blank feces and plasma were collected and then aspirin (37.5 mg·kg^−1^) was administered orally. The blood collection time was the same as that of the N1 group. Blank stools are used for microbiological testing and *in vitro* incubation experiment, and blood samples are used for pharmacokinetic experiments. Our research has been authorized by the Institutional Review Board of Lanzhou General Hospital of Lanzhou Military Command (Certificate of Approval: 201706030012). Animal experiments are carried out in accordance with the guidelines and regulations for laboratory animals established by our hospital.

### 16S rRNA analysis

Rats in the N1 and N2 groups were collected for feces (blank samples) before administration of aspirin, and six samples in each group were mixed two by two (*i*.*e*., three samples per group for DNA extraction). The DNA was extracted and then subjected to PCR according to the method reported in the literature^3,16^. The bioinformatics analysis will be carried out with sequencing data. The original data were filtered to remove low-quality information and the tags were clustered into operational taxonomic units (OTUs) with 97% sequence similarity. Taxonomic ranks were assigned to OUT emblematic list using Ribosomal Database Project (RDP) Naïve Bayes Classifier v.2.2. At last, alpha diversity, beta diversity, and the different species screening were analyzed.

### *In vitro* incubation experiment

To see if amoxicillin has an effect on intestinal metabolism of aspirin, the experiment was performed *in vitro*. The blank rat fecal specimens were weighed about 0.5 g and then suspended in 4.5 mL of cold saline. The fecal suspension was centrifuged at 5000 r·min^−1^ for 5 min. The resulting supernatant was sonicated for 10 min, then centrifuged at 100,000 r·min^−1^ for 20 min, and the supernatant was taken as a stool incubation solution. The incubation system consisted of 0.1 mL fecal solution, 0.1 mL of aspirin standard solution and 0.3 mL of phosphate buffer solution (0.1 M, pH 7.0), and incubated at 37 °C for 0, 12, 24, 36 h. After the end of the incubation, 9.5 mL of frozen acetonitrile was added to terminate the reaction.

### Plasma and stool sample processing

Accurately pipet 30 µL of plasma or fecal incubation solution into a 0.5 mL centrifuge tube, add 75 µL of acetonitrile (containing 1 µg·mL^−1^ of internal standard benzoic acid), vortex for 1 min, and centrifuge at 13000 r·min^−1^ for 5 min. The supernatant was taken in a sample bottle and the aspirin and salicylic acid were determined by liquid chromatography tandem mass spectrometry (LC-MS/MS). According to the measure condition reported in the literatures^17,18^, the specific analysis conditions we have established were as follows: acetonitrile-water-formic acid (90:10:0.1, v/v/v) was used as the mobile phase, and a single sample was analyzed for 3.0 min. The mass spectrometry spray voltage was −3500 V and the atomization temperature was 250 °C. The detection ion pairs of aspirin, salicylic acid and internal standard benzoic acid were *m/z* 178.8 → 137.0, *m/z* 136.8 → 93.0, *m/z* 120.8 → 77.0. The collision induced dissociation voltages were −11 psi, −26 psi, and −24 psi, and the collision energies were −13 eV, −26 eV, and −18 eV, respectively.

### Statistical analysis

The pharmacokinetic parameters of the aspirin metabolite salicylic acid in the two groups were calculated using DAS 2.0 software. Statistical differences between two groups were analyzed by the Student’s t-test using spss13.0 software, P < 0.05 had significant difference.
